# Water Area Extraction Using RADARSAT SAR Imagery Combined with Landsat Imagery and Terrain Information

**DOI:** 10.3390/s150306652

**Published:** 2015-03-19

**Authors:** Seunghwan Hong, Hyoseon Jang, Namhoon Kim, Hong-Gyoo Sohn

**Affiliations:** School of Civil and Environmental Engineering, Yonsei University, Seodaemun-gu, Seoul 120-749, Korea; E-Mails: hotaeim@yonsei.ac.kr (S.H.); hyoseon9026@yonsei.ac.kr (H.J.); knamsang@yonsei.ac.kr (N.K.)

**Keywords:** SAR sensors, thresholding method, object-based classification, flood mapping

## Abstract

This paper exploits an effective water extraction method using SAR imagery in preparation for flood mapping in unpredictable flood situations. The proposed method is based on the thresholding method using SAR amplitude, terrain information, and object-based classification techniques for noise removal. Since the water areas in SAR images have the lowest amplitude value, the thresholding method using SAR amplitude could effectively extract water bodies. However, the reflective properties of water areas in SAR imagery cannot distinguish the occluded areas caused by steep relief and they can be eliminated with terrain information. In spite of the thresholding method using SAR amplitude and terrain information, noises which interfered with users’ interpretation of water maps still remained and the object-based classification using an object size criterion was applied for the noise removal and the criterion was determined by a histogram-based technique. When only using SAR amplitude information, the overall accuracy was 83.67%. However, using SAR amplitude, terrain information and the noise removal technique, the overall classification accuracy over the study area turned out to be 96.42%. In particular, user accuracy was improved by 46.00%.

## 1. Introduction

SAR is an active sensor using a microwave signal, which can penetrate clouds and generate ground information regardless of the atmospheric conditions, unlike optics-based systems. Due to this characteristic, SAR can collect data from large areas under any weather conditions and the data is suitable for emergent disaster situations. In particular, when transmitted radar signals are reflected on flat water surfaces, a significantly weak return signal reaches the sensor and this characteristic makes it easy to identify flooding areas from SAR imagery [1,2]. Much research has been done on large scale flood mapping and flood dynamics [3,4,5,6,7,8,9,10,11,12]. In particular, the low return signal behavior of open water bodies supports the thresholding method [3,6,7,9,11,12]. For example, Schumann *et al.* [11] computed a threshold value using Otsu’s algorithm [13] to estimate the between-class variance from a normalized histogram. They used the Digital Elevation Model (DEM) of the Shuttle Radar Topography Mission (SRTM) and the ENVISAT imagery to estimate flood profiles on large rivers. Matgen *et al.* [12] set a radiometric threshold based on a gamma distribution assumption and combined it with a region growing approach to extract water bodies. 

Even though those histogram-based thresholding methods automatically obtain flood areas from SAR images with low complexity and computational efficiency, it is difficult to determine the optimized threshold. For example, the thresholding method only using SAR amplitude information cannot distinguish water from occluded regions caused by high terrain relief and flat land which has similar reflective characteristics with open water body [10,14]. Song *et al.* [10] used Gray Level Co-occurrence Matrix (GLCM), DEM and the Digital Slope Model (DSM) to remove the distortion caused by high relief and found that DSM displays the best performance in mountainous areas. Pierdicca *et al.* [15] integrated SAR imagery, land cover map and DEM into a fuzzy scheme for the flood mapping. Mason *et al.* [16] eliminated shadow and layover effects with airborne LiDAR data and detected urban flooding areas.

While the above methods can extract water bodies from SAR imagery, they have several limitations that impede their widespread use. Each flood mapping method with SAR imagery has just been studied in specific areas such as flat terrain, mountainous areas or urban areas. Because SAR reflectance properties are affected by the topography, vegetation and geometry of artificial objects, the signal return tendency cannot be fully estimated and the signal return tendency uncertainty decreases the performance of the water extraction using SAR imagery. For this reason, additional processes considering the ground conditions must be applied to the misclassified areas for large-scale flood mapping using SAR imagery. Moreover, since the pre-defined land cover information is required to determine the threshold value statistically, the periodic updating of land cover maps which have sufficient resolution and accuracy is a heavy and costly burden. Therefore, appropriate land cover maps which can be obtained instantly and a probabilistic approach to determine the threshold are required for practical water area mapping in unpredictable situations.

To overcome the abovementioned shortcomings of the existing methods, we proposed thresholding methods for water body extraction where threshold values concerning SAR amplitude and terrain information are determined based on the maximum-likelihood classifier and a land cover map created using Landsat TM imagery. We also applied the object-based algorithm to eliminate the misclassified pixels due to the unpredictable properties of land surfaces. For verification, we applied the proposed approach to RADARSAT-1 SAR images which captured the Jeollabuk-do area in Korea in 2005 when a flood event happened. A quantitative analysis was performed with a reference water map created using high-resolution orthorectified aerial images.

## 2. Methodical Background

### 2.1. Overview

**Figure 1:**
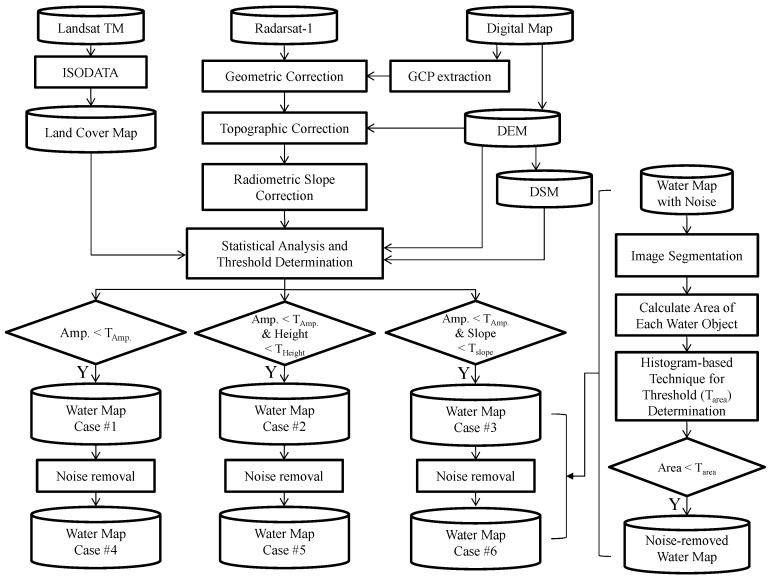
The schematic diagram of water area extraction.

The water extraction technique proposed in this study is divided into four steps as shown in Figure 1: (1) SAR image geocoding to ground coordinates and eliminating the geometric and radiometric topographic effect using DEM; (2) generation of a land cover map from Landsat TM imagery using the Interactive Self-Organizing Data Analysis (ISODATA) algorithm; (3) using the land cover map, determining threshold values for SAR amplitude and terrain information for water area classification; (4) eliminating the remaining noises using object-based classification with a histogram-based algorithm. 

Accurate geocoding is an important process for pixel-based image fusion [17]. The geocoding process includes three sub-procedures including obtaining Ground Control Points (GCPs), geometric correction and topographic correction. GCPs were obtained from 1:5000 scale digital topographic maps and the geometric correction is conducted using two fundamental equations in SAR geometry, *i.e.*, Range and Doppler equations [18]. Then, the topography correction was performed using DEM created from the 1:5000 scale digital topographic maps provided by the Korean National Geographic Information Institute. 

A land cover map is required to estimate the threshold values for the SAR amplitude and terrain information. For this purpose, we used Landsat 5 TM imagery with ISODATA algorithm to extract a land cover map of water and the other four classes including urban land, agricultural land, forest, rangeland, wetland and barren land and the classes has specific SAR amplitude and terrain properties. Among the five classes, threshold values of the SAR amplitude and terrain information were estimated using a maximum-likelihood classifier. The determined threshold value of SAR amplitude information could extract water bodies and that of terrain information could remove terrain areas distorted due to high relief. Even though most of water bodies could be extracted by the thresholding method, a number of misclassified objects decreased the accuracy. For this reason, object-based classification method based on the criterion about object sizes was conducted to eliminate unnecessary misclassified objects.

To verify the performance of the proposed method, we divided it into six cases and checked the accuracy of water area classification. We also focused on misclassified objects in high relief areas. The performance of the proposed method was assessed by producer accuracy corresponding to errors of commission and user accuracy corresponding to errors of omission. According to the input data and application of noise removal method, the six cases were defined as follows:

Case #1: Amplitude
Case #2: Amplitude + Height
Case #3: Amplitude + Slope
Case #4: Amplitude + Removal of the misclassified areas
Case #5: Amplitude + Height + Removal of the misclassified areas
Case #6: Amplitude + Slope + Removal of the misclassified areas

### 2.2. Geometric Correction and Topographic Correction of SAR Imagery

Since SAR imagery has geometric and radiometric distortion due to the platform and sensor information errors, topographic effects and atmospheric delay, geometric correction is indispensable for the pixel-based image fusion technique [10,19]. In particular, since the RADARSAT-1 satellite had provided inaccurate platform position and velocity information, an accurate geometric correction using sufficient GCPs is required. Substantial research to define rigorous 3-D physical models has been carried out and the mathematical functions for SAR imagery are the Doppler and Range equations, as shown in Equations (1) and (2) [18,20]:

(1) $$f=\frac{2(\vec{V_{S}}-\vec{V_{P}})(\vec{S}-\vec{P})}{\text{λ}\left|\vec{S}-\vec{P}\right|}$$

(2) $$r=\left|\vec{S}-\vec{P}\right|$$

where f is the Doppler value which is the difference between the Doppler centroid and Doppler shift, r is the range distance, $\vec{S}$ and $\vec{V_{S}}$ are the sensor position and velocity, $\vec{P}$ and $\vec{V_{P}}$ are the target point position and velocity on the ground, and λ is the radar wavelength. The Doppler and Range equations are solved simultaneously to correct orbit parameters which are definition can be represented by quadratic time-dependent equations of sensor position and velocity as follows:

(3) $$\vec{S}=\left[\begin{array}{c} a_{0}+a_{1}t+a_{2}t^{2}\\ b_{0}+b_{1}t+b_{2}t^{2}\\ c_{0}+c_{1}t+c_{2}t^{2} \end{array}\right],{\vec{V}}_{s}=\left[\begin{array}{c} d_{0}+d_{1}t+d_{2}t^{2}\\ e_{0}+e_{1}t+e_{2}t^{2}\\ f_{0}+f_{1}t+f_{2}t^{2} \end{array}\right]$$

where, t is the pixel sampling timing and $a_{0},a_{1},a_{2},b_{0},...,f_{0},f_{1}\;and\;f_{2}$ are orbit parameters which are geometrically corrected.

Even after the geometric correction, topographic distortion is still remained because the terrain relief causes geometric terrain distortions such as overlay, foreshortening and shadow effects, and thus could produce positional errors and make interpretation of SAR imagery difficult. Not only that, but due to the local incidence angle between the SAR sensor and the actual terrain, the actual back scattering is different from that estimated based on a flat Earth assumption [10,18]. In SAR imagery, the radiometric effect of non-flat scattering varies according to the ground slope, incidence angle and the azimuth direction and the power of received waves from the ground target point is represented by Equation (4) [21]:

(4) $$P_{r}=K_{A}*\frac{P_{t}\lambda^{2}G_{t}(\gamma )G_{r}(\gamma )}{{\left(4\pi \right)}^{3}R^{4}}\sigma^{0}A$$

where, $P_{t}$ is the transmitted power; λ is the radar wavelength, R is the distance to the scattering area, γ is the radar look angle, $G_{t}(\gamma )$ and $G_{r}(\gamma )$ are the transmitted and received antenna gains at look angle γ respectively, and ${\text{σ}}^{0}$ is the normalized radar cross section for area A. $K_{A}$ is correction coefficient for topographic effect and defined as in Equation (5):

(5) $$K_{A}=A’/A$$

with:

(6) $$\;A’=\frac{\delta_{r}}{\sin(\eta -\varphi_{r})}\frac{\delta_{a}}{\cos(\varphi_{a})}$$

(7) $$A=\frac{\delta_{r}\delta_{a}}{\sin(\eta )}$$

where A is the flat ground area without terrain relief or spherical Earth and A’ is the actual scattering area of the non-flat terrain. η is the incidence angle, ${\text{δ}}_{r}$ and ${\text{δ}}_{a}$ are the slant range and azimuth pixel spacing, respectively, and ${\text{φ}}_{r}$ is the tilt of the surface in the range direction, and ${\text{φ}}_{a}$ is the tilt of the surface in the azimuth direction. The accurate information of SAR signal scattering surface is required to correct the topographic effect caused by local terrain relief and can be calculated from the geometry between DEM and satellite orbit model.

### 2.3. Threshold Determination

The maximum-likelihood classifier is one of the most widely used supervised classification methods [22]. The supervised classification method needs appropriate training data to estimate threshold values, however, it is difficult to achieve suitable reference data like a land cover map when the water map is needed in unpredictable situations like floods. For this reason, cloud-free Landsat TM imagery captured on the closest date to the SAR imagery was utilized to create a land cover map. Officially, the land cover maps made by the Korean Ministry of Environment divide the territory in Korea into seven classes: urban land, agricultural land, forest, rangeland, wetland, barren land and water. Because our purpose is the extraction of water area and flood detection using SAR imagery and terrain information, the land cover map with water class and the other four classes including the classes of urban land, agricultural land, forest, rangeland, wetland and barren land is enough to create a water map. This is because that agricultural land, rangeland and wetland have very similar SAR amplitude and terrain information properties.

In maximum-likelihood classification, each pixel is assigned a certain class i which has the highest probability $\left(p_{i}(X)=p(X|C_{i})\cdot p(C_{i})\right)$. Since the prior probability $\left(p(C_{i})\right)$ typically cannot be estimated, it is assumed that the prior probabilities of all classes are equal. When two classes have the same probability to be included, the value of threshold (T) is defined by Equation (8):

(8) $$T=\{X|p_{i}(X)=p_{j}(X)\}$$

However, the equation $p_{i}(X)=p_{j}(X)$ does not guarantee that the threshold value will be uniquely defined [23]. In this study, we used a threshold value between the two mean values of classes. Since the reflectance characteristics of homogeneous areas in SAR images including speckle noise typically determined by gamma distribution [24], the gamma probability density function was used to estimate threshold values on the SAR amplitude. The gamma distribution can be applied to positive values. To shift the entire amplitude values to positive values, Matgen *et al.* [12] added minimum amplitude value to entire values and the equation of the gamma probability density function is given as follows:

(9) $$p_{i}(x/k_{i},\theta_{i})=\frac{{\left(x-x_{\min}\right)}^{k_{i}-1}}{\theta^{k_{i}}\Gamma (k_{i})}e^{\frac{\left(x-x_{\min}\right)}{\theta_{i}}}$$

where, x is the pixel value of SAR imagery, $x_{\min}$ is the minimum pixel value of SAR imagery, $k_{i}$ and ${\text{θ}}_{i}$ are the shape parameter and the scale parameter of class i and $\Gamma (k_{i})$ is the gamma function about $k_{i}$. In case of the threshold determination about terrain information, the normal distribution was used and the normal probability density function of i is represented by Equation (10):

(10) $$p_{i}(x/\mu_{i},\sigma_{i})=\frac{1}{\sigma_{i}\sqrt{2\pi }}e^{-\frac{{\left(x-\mu_{i}\right)}^{2}}{2{\sigma_{i}}^{2}}}$$

where, ${\text{μ}}_{i}$ and ${\text{σ}}_{i}$ are mean and standard deviation of class i.

There were four threshold values each relating to SAR amplitude and terrain information accordingly as the water class and the other four classes were in the land cover map. Of the SAR amplitude information and terrain information, the highest value among the estimated values was respectively selected for the threshold value. In the case of water extraction using a threshold for SAR amplitude information, low threshold values could misclassify actual water bodies as land. For the threshold value relating to terrain information, the occluded areas caused by steep relief have low values like water areas and the most of the steep relief exists on mountainous areas at a high altitude and with a high slope. For this reason, the selected threshold values relating to SAR amplitude and terrain information can extract water bodies from the occluded area with minimized misclassification.

### 2.4. Noise Removal

Threshold taxonomy is used on SAR imagery for water area classification in order to effectively enhance the classification accuracy in the mountainous region areas, but removal of the misclassified objects which appear in the form of noise in the image is required. Since the misclassified segments appear like small points, these types of error can be removed based on the size of extracted objects. The object size criterion to distinguish the misclassified objects is determined by the histogram-based technique proposed by Zack *et al.* [25]. It is an algorithm that searches for a valley point of the histogram corresponding to the farthest point on the line connecting the value of highest peak and the maximum value in the histogram. The advantage of this method is unaffected by histogram irregularities for valley detection. The algorithm can be described by Equations (11)–(15) [26]. The model of the straight line connecting two points $P_{1}(A_{1},f_{1})$ and $P_{2}(A_{2},f_{2})$ is represented by Equation (11):

(11) a⋅x+b⋅y+c=0

with:

(12) $$a=\frac{f_{1}-f_{2}}{A_{1}-A_{2}}$$

(13) b=-1

(14) $$c=f_{1}-a\cdot A_{1}$$

The distance between each point $P_{i}$ on the graph to the line is calculated by Equation (15):

(15) $$D_{p_{i}}=\left|\frac{\;a\cdot x_{p_{i}}+b\cdot y_{p_{i}}+c}{\sqrt{a^{2}+b^{2}}}\right|$$

The histogram valley corresponds to the point of the histogram whose distance $D_{P_{i}}$ is farthest.

## 3. Experiments and Results

### 3.1. Study Sites and Data Preparation

**Figure 2:**
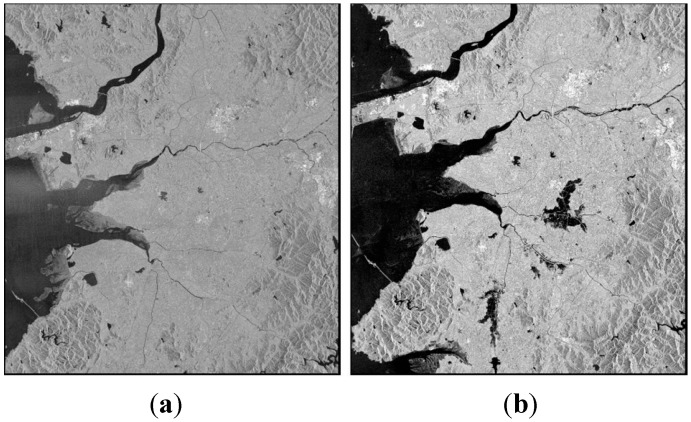
RADARSAT-1 SAR images acquired over the study site area: (**a**) Not flooded; (**b**) Flooded.

**Figure 3:**
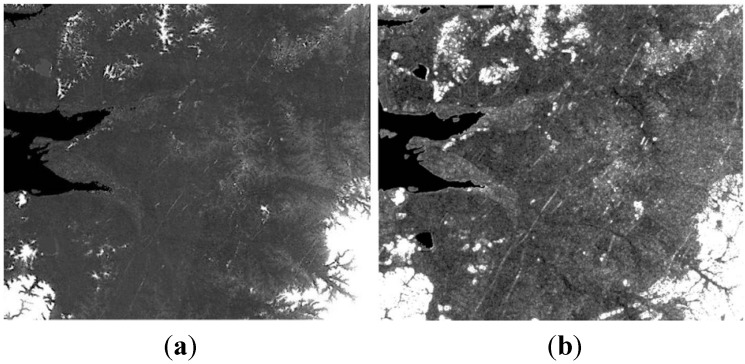
Terrain information: (**a**) DEM; (**b**) DSM.

Test image scenes were obtained from RADARSAT-1 SAR imagery as shown in Figure 2 to investigate the suitability of the proposed flood mapping procedure. These two images clearly show the pre-post differences in the flooded areas caused by the disaster in 2005. Images captured in fine mode with about 40° incidence angle and 6.25 m spatial resolution were used. The study area covers the Jeollabuk-do region of Korea in which flooding damage occurred during July to August of 2005 due to heavy rainfall. This flood not only caused five times larger recovery costs than average but also killed 12 people and injured 26 people. The maximum elevation of the test site is 605 m, and the maximum terrain slope is up to 87.24°. Figure 3 shows the DEM and DSM which are the terrain information for geometric correction. The maximum change of elevation over the pixel distance in the 3 × 3 window defined the slope values in the DSM. The DEM whose spatial resolution is 5 m was produced using digital aerial images and the accuracy was verified by ground survey. DSM was generated by the DEM.

**Figure 4:**
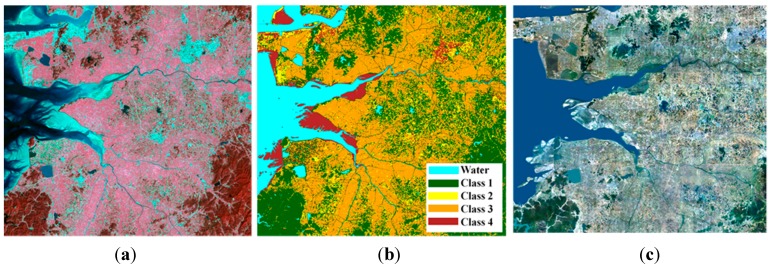
(**a**) Landsat TM imagery; (**b**) land cover map created using Landsat imagery (In general, class 1 represented forested land, class 2 was urban land and class 3 contained agricultural land, rangeland and wetland, and barren land and tideland consisted of class 4); (**c**) orthorectified aerial image over the study area.

Figure 4 is Landsat TM imagery, the land cover map created using ISODATA algorithm and an orthorectified aerial image over the study area. The cloud-free Landsat imagery was acquired on 21 September 2003 was the closest to the flood epoch. The ISODATA algorithm was carried out to classify water and the other four class types. All of the data were resampled at 6.25 m pixel-spacing using a nearest neighborhood interpolation to be co-registered with the fine mode SAR images. The reference water map for the accuracy assessment was manually extracted from the orthorectified digital color aerial images which have 50 cm spatial resolution and 25 cm geolocation accuracy.

### 3.2. Geometric Correction of SAR Images

**Figure 5:**
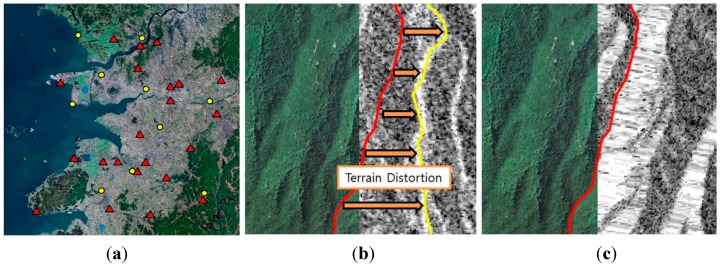
GCPs used for geometric correction and result of geometric and radiometric topographic correction: (**a**) GCPs (yellow: Control point, red: Check point, blue: reference water map); (**b**) orthorectified aerial image and SAR imagery before terrain effect correction (red: an actual ridge, yellow: a ridge represented in SAR imagery, orange: inconsistency of ridge location due to terrain distortion); (**c**) orthorectified aerial image and SAR imagery after terrain effect correction (red: an actual ridge corresponds with a ridge represented in SAR imagery).

There is geometric and radiometric terrain distortion due to topographic properties as well as inaccurate platform positional data which the RADARSAT-1 satellite provided. To eliminate the positional error of the RADARSAT-1 imagery and alleviate the terrain distortion, 10 GCPs and 15 check points in the each image were selected from 1:5000 Korean digital topographic maps and orthorectified digital aerial images. The 10 GCPs were used to refine the orbit parameters and the precision of the correction was checked by the 15 check points. As a result of the geometric correction, the precisions of two SAR images were ensured within 0.5 pixels of RMSE. Figure 5 shows the selected GCPs and check points in the 50 cm spatial resolution orthorectified digital aerial images and the topographic correction results. Before topographic effect correction, there was significant distortion between the actual locations of mountain ridges and ridges represented in the SAR imagery as shown in Figure 5b. After topographic correction using DEM generated from digital topographic map, as shown in Figure 5c, the topographic effects such as overlay and foreshortening were significantly alleviated.

### 3.3. Statistics and Threshold of SAR Amplitude and Terrain Information

Table 1 and Table 2 are the statistics and the thresholds for SAR amplitude and terrain information, which were estimated for water and the other four land cover classes. As shown in Table 1 and Table 2, Class 1 had the properties of relatively high amplitude value, altitude and steep slope. Class 1 represented forested land, which occupies 70% of the Korean territory. Class 2 was urban land and Class 3 contained agricultural land, rangeland and wetland. Classes 2 and 3 had higher SAR amplitude, altitude and slope values than the water class, but the altitude and slope values were lower than for Class 1. Class 4 consisted of barren land and tideland. Since the tideland contained a large amount of moisture, its SAR amplitude value was relatively lower than all the other classes, except for the water class.

Using statistical properties and a maximum-likelihood classifier, we calculated the threshold values between the water class and the other classes. Since it is ambiguous to determine an optimized threshold, the determination of a suitable value is one of the most important issues for any thresholding technique. If too low a threshold value is selected, water pixels in SAR imagery can be classified as land. Therefore, the highest value of the estimated SAR amplitude thresholds was selected. Class 2 and Class 3 had the highest decision boundary value which can extract water bodies from SAR imagery.

In the case of topographic properties, the threshold of terrain information between the water class and Class 1 was selected to eliminate the terrain distortion. This is because Class 1 which contains mountainous areas has high altitude and steep slope properties which cause occluded areas. The threshold value relating to topographic properties could eliminate occluded areas due to high relief.

**Table 1:** Statistics and SAR amplitude threshold values (08.03/08.23).

Class	Mean	Std. Dev.	Max	Min	T_amplitude_
Class 1	−8.02/−7.80	6.48/6.25	15.13/15.00	−76.83/−80.95	−14.86/−13.10
Class 2	−6.55/−6.59	6.53/6.28	15.08/14.63	−76.81/−70.78	−14.25/−12.55
Class 3	−7.32/−7.04	5.79/4.79	14.80/14.55	−77.41/−79.52	−14.44/−12.68
Class 4	−11.95/−9.78	8.44/6.87	14.81/14.86	−87.77/−84.99	−15.77/−13.66
Water	−20.83/−17.79	5.27/5.40	14.50/14.92	−90.01/−84.96	-

**Table 2:** Statistics and terrain information threshold values (DEM/DSM).

Class	Mean	Std. Dev.	Max	Min	T_topographic_
Class 1	54.60/8.66	80.29/10.68	605.00/87.24	0/0	17.72/2.88
Class 2	15.80/2.33	17.03/4.02	447.32/71.37	0/0	8.35/0.67
Class 3	8.80/0.80	14.91/2.67	573.13/66.97	0/0	4.55/-
Class 4	6.89/1.05	12.45/3.57	413.43/75.10	0/0	3.15/-
Water	2.55/0.23	13.72/2.42	513.66/77.20	0/0	-

**Figure 6:**
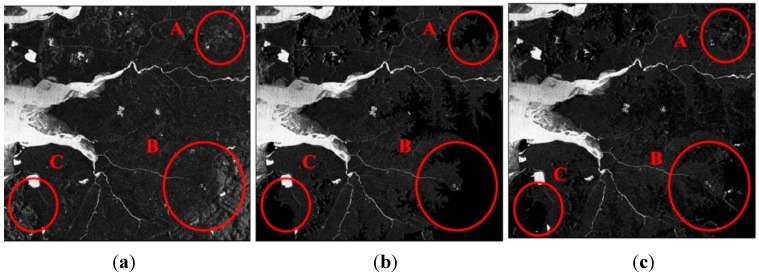
The water classification results of: (**a**) Case #1; (**b**) Case #2; (**c**) Case #3.

Table 3 and Figure 6 are the classification result and the A, B and C regions in Figure 6 are the areas having high relief. As shown in the results, the misclassified region caused by the steep relief could be eliminated effectively using the slope threshold. Although elevation information was also able to get rid of the misclassified pixels, most water bodies in high lands were removed by the elevation threshold. In the case where only the amplitude value was used, the overall accuracy was 83.67%. When removing the occluded regions by using DEM and DSM, 88.93% and 88.81% overall accuracies were confirmed, respectively. The accuracies of Case #2 and Case #3 were higher than that of Case #1. As shown in region B, the elevation information removes more misclassified objects in high land than slope information, therefore, the accuracy of Case #2 seemed a little better than Case #3. However, the DEM threshold got rid of reservoirs and streams in the high land. Focusing on the A, B and C regions in Figure 6, while the elevation information of Case #2 removed not only occluded regions but also water objects at high altitude, the slope information of Case #3 only eliminated occluded areas except for water bodies in mountainous areas.

**Table 3:** The water classification results using terrain information.

Case	Producer Accuracy (%)		User Accuracy (%)		Overall Accuracy (%)
	Water	Non-Water	Water	Non-Water	
1	83.00	83.80	48.54	96.40	83.67
2	81.57	90.28	60.72	96.38	88.93
3	82.66	89.94	60.21	96.57	88.81

Cases #1: Amplitude, Cases #2: Amplitude + Elevation, Cases #3: Amplitude + Slope.

### 3.4. Object-Based Noise Removal

**Figure 7:**
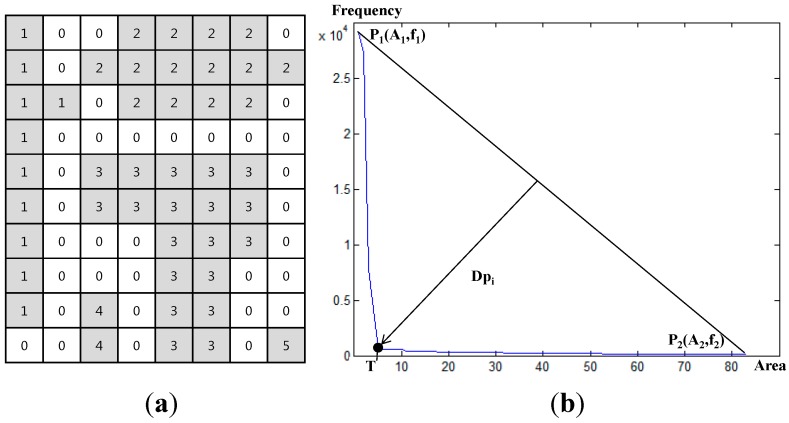
(**a**) Example of labelled objects (Gray: water, White: non-water); (**b**) Determination of threshold to remove the misclassified objects.

After thresholding with respect to SAR amplitude and terrain information, the water map could be represented by millions of labelled objects. Figure 7 is an example of labelled objects and application of the algorithm to the actual SAR imagery captured in 3 August. The objects were sorted by the object size and drawn on the histogram in order to estimate the threshold (T) to get rid of the misclassified objects. As shown in Figure 7b, the valley of this histogram was evidently visible and it was easily calculated by the algorithm mentioned in Section 2.4.

**Figure 8:**
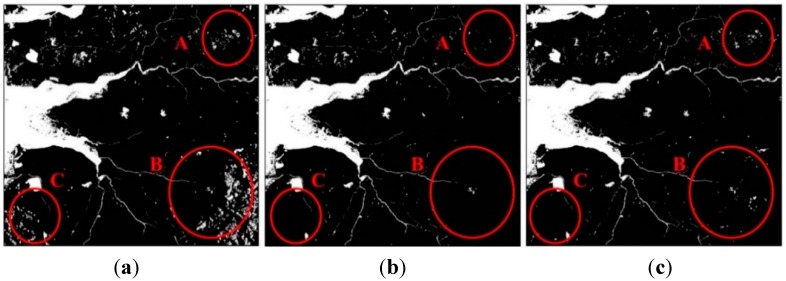
The water classification results of: (**a**) Case #4; (**b**) Case #5; (**c**) Case #6.

Table 4 and Figure 8 are the result of the noise removal. After differentiating the misclassified segments, the accuracies of Cases #4, #5 and #6 were greatly improved and the accuracies were 93.93%, 96.22% and 96.42%, respectively. As a result of Case #4, we found that the object segmentation method without terrain information was not able to remove the misclassified objects due to steep relief. In particular, the user accuracy for water extraction was remarkably improved to 94.54% compared to 48.54% in Case #1.

**Table 4:** The water classification results after noise removal.

Case	Producer Accuracy (%)		User Accuracy (%)		Overall Accuracy (%)
	Water	Non-Water	Water	Non-Water	
4	81.65	96.19	79.78	96.61	93.93
5	80.69	99.07	94.14	96.54	96.22
6	81.69	99.13	94.54	96.71	96.42

Cases #4: Amplitude + Removal of the misclassified, Cases #5: Amplitude + Elevation + Removal of the misclassified, Cases #6: Amplitude + Slope + Removal of the misclassified.

### 3.5. Flood Mapping

**Figure 9:**
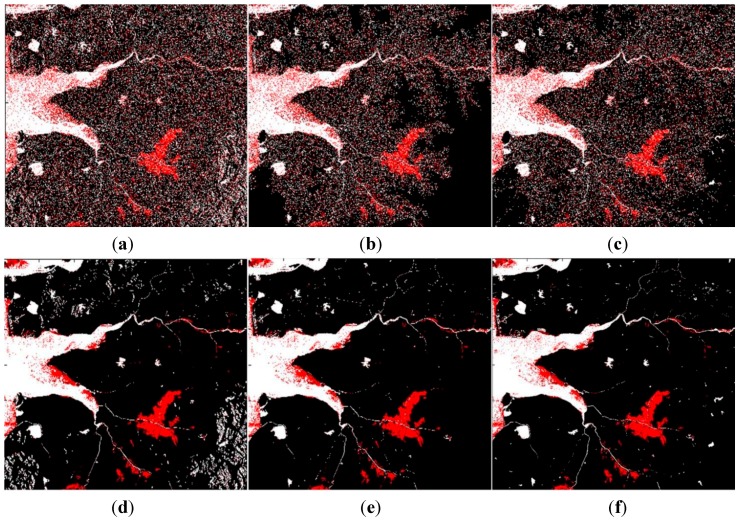
Flood map (red: flooded areas, white: permanent water areas): (**a**) Case #1; (**b**) Case #2; (**c**) Case #3; (**d**) Case #4; (**e**) Case #5; (**f**) Case #6.

Figure 9 illustrates the flood mapping results. The red parts in the figures were the detected flood areas during the flooding season, and white regions are permanent water bodies in the reference map. Comparing the flood mapping results, we found that it was impossible to create accurate flood maps in mountainous regions without slope information and flood maps using the elevation information were not substantially helpful to eliminate the topographic distortion. As shown in the results of Cases #4, #5 and #6, the object-based technique proposed in this paper could remove the misclassified objects remarkably well and the flood map of Case #6 showed the clearest result.

**Figure 10:**
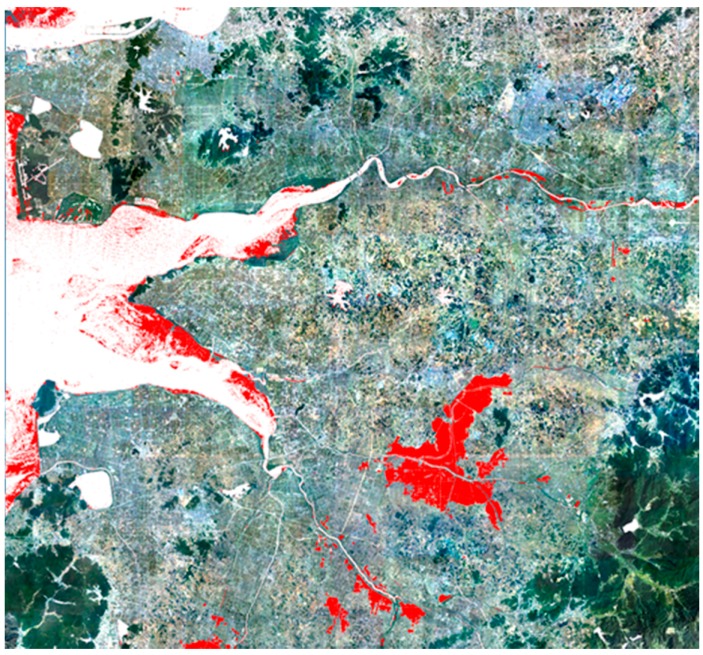
Map of flood area (red: flooded areas, white: permanent water areas).

Figure 10 is the flood map overlapped on the orthorectified aerial imagery. As shown in Figure 10, the flooded areas are clearly represented without noises.

## 4. Conclusions

Estimating optimized threshold values for water body extraction and the removal of misclassified land objects are key issues for water mapping using SAR imagery. This paper proposed water extraction methods using SAR amplitude imagery and terrain information such as DEM, DSM for the thresholding method and object-based noise removal method.

Prior to applying the proposed method, we corrected the satellite parameters and removed the topographic distortion for locational consistency of input data. Then, a land cover map which classified water and four other classes of land was created from Landsat TM imagery using the ISODATA algorithm and used to determinate threshold values. The estimated threshold value relating to SAR amplitude could find water areas due to the reflective properties of water in SAR imagery, however, there were occluded areas due to steep relief and the slope information could remove the misclassified areas. Because eliminating occluded areas using the elevation information tended to erase water areas at high altitude, the slope information showed better performance for this problem. Even if the thresholding method using SAR amplitude and terrain information was applied to extract water bodies, noises which were non-water areas classified as water areas remained and it reduced the user accuracy of the water map. The object-based classification method with an object size criterion was applied to remove the noises. The criterion was estimated with a histogram-based technique. With the object-based method, the noise objects were eliminated and the classification accuracy was significantly improved. In particular, the user accuracy was remarkably improved.

In this study, the proposed water classification procedure was applied to medium resolution SAR images (C band, 6.25 m of spatial resolution) and it could solve the problems effectively. The limitation of the proposed method is the resolution and quality of the input data. Even though low resolution multi-spectral imagery (Landsat TM, 30 m of spatial resolution) and a digital map with 2.5 m of height accuracy were used, the proposed method could classify the water areas from SAR imagery with high accuracy. However, to apply the proposed method for high resolution SAR data, more accurate and detailed elevation and land cover information is necessary, but construction of high-quality data is costly and time-consuming, therefore, the problem of how to overcome the limitations of ancillary data quality requires further work.
